# Workplace factors associated with willingness to undergo human immunodeficiency virus testing during workplace health checkups

**DOI:** 10.1265/ehpm.23-00054

**Published:** 2023-09-22

**Authors:** Kazuyoshi Mizuki, Tomohiro Ishimaru, Mayumi Imahashi, Yuzuru Ikushima, Hideto Takahashi, Masashi Masuda, Yoshiyuki Yokomaku

**Affiliations:** 1 Department of Environmental Epidemiology, Institute of Industrial Ecological Sciences, University of Occupational and Environmental Health, Japan, Kitakyushu, Japan; 2 Department of Medical Humanities, School of Medicine, University of Occupational and Environmental Health, Japan, Kitakyushu, Japan; 3 Department of Infectious Diseases & Immunology, Clinical Research Center, Nagoya Medical Center, Nagoya, Japan; 4 Place Tokyo, Tokyo, Japan; 5 National Institute of Public Health, Wako, Japan

**Keywords:** AIDS, HIV, Japan, Workers, Testing

## Abstract

**Background:**

To examine workplace factors associated with willingness to undergo human immunodeficiency virus (HIV) testing during workplace health checkups.

**Methods:**

This cross-sectional study used an Internet-based self-administered questionnaire to obtain data from a pool of 24,287 Japanese workers. Binary and multiple logistic regression analyses evaluated the association between workplace factors and HIV testing. Data were adjusted for sex, age, marital status, education, and history of HIV testing.

**Results:**

We gathered information from 4,143 (17.1%) respondents, of whom 1,129 (27.3%) were willing to be tested for HIV as part of a workplace health checkup. The participants were 20–59 years old. Approximately half of the participants were male (49.9%), half were married (48.9%), and half had completed higher education (47.6%). Workplace hepatitis testing was offered to 15.6% of the respondents, and most participants underwent health checkups without their colleagues (52.1%) at a medical facility (60.2%). Willingness to undergo HIV testing was positively correlated with having an increased risk of occupational blood exposure (vs. not at risk, adjusted odds ratio [OR]: 1.74, 95% confidence interval [CI]: 1.41–2.15) or working in medical and welfare roles (vs. manufacturing, OR: 1.40, 95% CI: 1.07–1.84). The presence of occupational health staff at the workplace (vs. their absence, adjusted OR: 1.35, 95% CI: 1.16–1.59) and hepatitis testing (vs. not testing, adjusted OR: 2.02, 95% CI: 1.66–2.44) increased willingness to undergo HIV testing.

**Conclusions:**

A pilot HIV-testing program involving individuals at an increased risk of occupational blood exposure and undergoing hepatitis tests in workplaces providing occupational health staff support is recommended.

## Background

Opportunities for human immunodeficiency virus (HIV) screening should be increased, because early diagnosis and treatment can improve patients’ quality of life [1]. Recently, the annual number of HIV tests being performed in Japan has decreased dramatically from 142,260 in 2019 to 58,172 in 2021 [2]. This decrease is likely to have occurred because of waning interest in HIV as a result of the coronavirus disease 2019 (COVID-19) pandemic [3]. In the United States (US), where the prevalence of HIV is relatively high, HIV testing is recommended for all patients aged between 13 and 64 years [4]. In contrast, in Japan, where the prevalence of HIV is low, HIV testing has been recommended for high-risk populations such as men who have sex with men, commercial sex workers, and people who inject drugs [5]. However, there is concern that the number of HIV-infected people in Japan may increase in the future for the following reasons: 1) the detection rate of acquired immunodeficiency syndrome (AIDS) in patients previously unaware of their HIV-positive status (known as Ikinari-AIDS) in Japan remains high (∼30%) [2]; and 2) the number of people with syphilis is the highest reported in decades [6]. A previous study has reported a high likelihood of simultaneous transmission of HIV and syphilis [7].

Providing HIV testing during health checkups in the workplace could effectively increase the rate of HIV testing and help to control the spread of HIV infection. In a previous Japanese study, 41% of workers were willing to be tested for HIV during workplace health checkups [8]. In Japan, the Ministry of Health, Labour, and Welfare is already promoting virus testing during workplace health checkups and started testing for hepatitis B/C virus in 2011 [9] and for rubella antibodies in 2019 [10, 11]. As a result of this drive to increase testing, many people have undergone the hepatitis B/C virus [12] and rubella antibody screening tests [13]. Previous studies have reported that receiving accurate information and understanding government policies influenced the willingness of individuals to take these screening tests [9, 10].

Identifying the factors that encourage people to undergo HIV testing in the workplace is important for incorporating HIV testing into workplace health checkups. Fear of discrimination and stigma, which are associated with the sensitive nature of the test results, discourages individuals from getting HIV-tested [14–16]. Conversely, the risk perception of HIV infection and education about HIV increase HIV-testing compliance [17–20]. However, few studies have evaluated the association between workplace factors (such as occupation type, company structure, or implementation of workplace health checkups) and the willingness to undergo HIV testing during workplace health checkups. The purpose of the current study was to examine workplace factors associated with willingness to undergo HIV testing during workplace health checkups.

## Methods

### Study design and participants

This cross-sectional study was conducted using an Internet-based self-administered questionnaire. The questionnaire was administered by an Internet research company (Intage Inc., Tokyo, Japan), which has access to approximately 3.21 million registered users who actively engage in a diverse range of surveys. For the current study, 24,287 people were randomly selected and sent a preliminary questionnaire via email in December 2021. Informed consent was obtained before the participants gave their responses, and only the responses of those who consented were analyzed. Among Japanese workers aged 20–59 who were identified through screening with a preliminary questionnaire, a stratified sampling method was used to ensure equal sex and age ratios in each group. Recruitment was completed after we collected data from approximately 500 participants in each stratum. Age groups were stratified as follows: 20–29, 30–39, 40–49, and 50–59 years. Participation was strictly voluntary, and the collected data were kept anonymous. Participants received a financial incentive from the Internet research company in the form of electronic points that could be exchanged for products worth several US dollars. The study was approved by the Ethics Committee of the University of Occupational and Environmental Health, Japan (R3-052).

### Questionnaire outcome

In our previous study [8], we asked participants about their willingness to undergo HIV testing during workplace health checkups using the following question: “Do you want to have a blood test for HIV screening as part of a health checkup in the workplace if it is free of charge?” The response options were: “yes,” “no,” and “unknown.” In this analysis, “unknown” was classified as “no” to divide the responses into binary “yes” and “no” data.

### Workplace factors

Through discussions with an expert panel of HIV specialists, occupational health practitioners, support group staff, and epidemiologists, as well as a review of the relevant literature, we identified three workplace factors that could potentially influence willingness to undergo HIV testing during workplace health checkups: (1) type of occupation (two items); (2) company structure (two items); and (3) implementation of workplace health checkups (three items). The “type of occupation” factored in the risk of occupational blood exposure and the type of industry. The industry category was based on the Japan Standard Industrial Classification of the Ministry of Internal Affairs and Communications [21]; industries associated with <2% of respondents were classified as “other.” We asked about workplace structure: the presence of occupational health staff in the workplace, and the size of the company. Company size was divided into four categories according to the total number of employees: micro scale (<10 employees), small scale (10–49 employees), medium scale (50–999 employees), or large scale (≥1000 employees). The “implementation of workplace health checkups” included voluntary hepatitis testing during workplace health checkups, health checkups with colleagues, and the location of health checkups. The options for the location of health checkups were: “workplace,” “medical institution,” and “other.”

### Covariates

We asked about age, sex, marital status, education, and history of HIV testing. The response to a history of HIV testing was: “yes,” “no,” and “unknown.” Respondents who indicated having a history of HIV testing selected the time of their last HIV test from the following options: “within 1 year,” “1–3 years,” “>3 years,” and “unknown.”

### Data analysis

Binary and multiple logistic regression analyses were used to assess the association between workplace factors and willingness to undergo HIV testing during workplace health checkups. In the multivariate model, we adjusted for sex, age, marital status, education, and history of HIV testing. Stata/SE 16.1 software (StataCorp, College Station, TX, USA) was used for data analysis. All *P*-values were two-sided and considered to indicate statistical significance if <0.05.

## Results

A total of 4,143 participants responded to the survey, with a valid response rate of 17.1%. Table 1 shows participants’ demographic characteristics. Of the participants, 1,129 (27.3%) were willing to be tested for HIV as part of workplace health checkups. Each age group was approximately equally represented, with approximately half of the participants being male (49.9%). Married individuals (48.9%) and people whose highest level of education was university or graduate school (47.6%) were the most prevalent within the study population. There was a history of HIV testing in 11.7% of the participants. Regarding work type, 11.8% of the respondents were at risk of occupational blood exposure, and the most common industry was manufacturing (17.0%), followed by retail and wholesale (11.8%), and medical and welfare (11.2%). In terms of company structure, occupational health staff were present in one-third of the workplaces (34.1%) and the most typical company size was medium scale (35.9%). Regarding the implementation of workplace health checkups, access to hepatitis testing was available to 15.6% of the respondents. Most participants did not have health checkups with their colleagues (52.1%) and the majority of the health checkups (60.2%) were performed in a medical facility.

**Table 1:** Demographic characteristics of the participants

	**Total**	**Willingness to undergo voluntary HIV testing during health checkups**	

	**N = 4,143**	**Yes** **n = 1,129** **(27.3%)**	**No** **n = 3,014** **(72.7%)**

	**n (%)**	**n (%)**	**n (%)**
Age			
20–29 years	1,026 (24.8)	357 (31.6)	669 (22.2)
30–39 years	1,033 (24.9)	342 (30.3)	691 (22.9)
40–49 years	1,048 (25.3)	247 (21.9)	801 (26.6)
50–59 years	1,036 (25.0)	183 (16.2)	853 (28.3)
Sex			
Women	2,076 (50.1)	536 (47.5)	1,540 (51.1)
Men	2,067 (49.9)	593 (52.5)	1,474 (48.9)
Marital status			
Single	1,828 (44.1)	506 (44.8)	1,322 (43.9)
Divorced or widowed	290 (7.0)	82 (7.3)	208 (6.9)
Married	2,025 (48.9)	541 (47.9)	1,484 (49.2)
Education			
Junior high or high school	1,203 (29.0)	299 (26.5)	904 (30.0)
Vocational school or college	969 (23.4)	245 (21.7)	724 (24.0)
University or graduate school	1,971 (47.6)	585 (51.8)	1,386 (46.0)
History of HIV testing			
No	3,541 (85.5)	850 (75.3)	2,691 (89.3)
Unknown	117 (2.8)	33 (2.9)	84 (2.8)
Yes	485 (11.7)	246 (21.8)	239 (7.9)
<1 year	85 (2.1)	62 (5.5)	23 (0.8)
1–3 years	93 (2.2)	58 (5.1)	35 (1.2)
>3 years	291 (7.0)	122 (10.8)	169 (5.6)
Unknown	16 (0.4)	4 (0.4)	12 (0.3)
Risk for occupational exposure to blood			
No	3,577 (86.3)	910 (80.6)	2667 (88.5)
Unknown	78 (1.9)	16 (1.4)	62 (2.0)
Yes	488 (11.8)	203 (18.0)	285 (9.5)
Industry			
Manufacturing	704 (17.0)	192 (17.0)	512 (17.0)
Retail and wholesale	488 (11.8)	122 (10.8)	366 (12.1)
Medical and welfare	463 (11.2)	162 (14.3)	301 (10.0)
Transportation	237 (5.7)	56 (5.0)	181 (6.0)
Information technology	221 (5.3)	72 (6.4)	149 (4.9)
Public service	208 (5.0)	77 (6.8)	131 (4.3)
Education and learning support	206 (5.0)	53 (4.7)	153 (5.1)
Construction	190 (4.6)	41 (3.6)	149 (4.9)
Finance	163 (3.9)	47 (4.2)	116 (3.8)
Catering and hospitality	156 (3.8)	41 (3.6)	115 (3.8)
Living-related services and entertainment	155 (3.7)	43 (3.8)	112 (3.7)
Other	952 (23.0)	223 (19.8)	729 (24.2)
Occupational health staff in the workplace			
No	2,208 (53.3)	559 (49.5)	1,649 (54.7)
Unknown	524 (12.6)	107 (9.5)	417 (13.8)
Yes	1,411 (34.1)	463 (41.0)	948 (31.5)
Company size			
1–9 employees	697 (16.8)	159 (14.1)	538 (17.9)
10–49 employees	818 (19.8)	235 (20.8)	583 (19.3)
50–999 employees	1,489 (35.9)	395 (35.0)	1,094 (36.3)
≥1000 employees	1,139 (27.5)	340 (30.1)	799 (26.5)
Voluntary hepatitis testing during workplace health checkups			
No	2,665 (64.3)	674 (59.7)	1,991 (66.1)
Unknown	834 (20.1)	176 (15.6)	658 (21.8)
Yes	644 (15.6)	279 (24.7)	365 (12.1)
Health checkups with colleagues			
No	2,160 (52.1)	513 (45.4)	1,647 (54.7)
Unknown	191 (4.6)	37 (3.3)	154 (5.1)
Yes	1,792 (43.3)	579 (51.3)	1,213 (40.2)
Location of health checkups			
Workplace	1,223 (29.5)	329 (29.1)	894 (29.7)
Medical facility	2,495 (60.2)	713 (63.2)	1,782 (59.1)
Other	425 (10.3)	87 (7.7)	338 (11.2)

HIV: human immunodeficiency virus.

Table 2 shows the results of the logistic regression analysis of workplace factors associated with the willingness to undergo HIV testing during workplace health checkups. In terms of the type of occupation, strong positive correlations were observed between the willingness to undergo HIV testing and having an occupation that placed workers at risk of occupational blood exposure (compared with those not at risk, adjusted odds ratio [OR]: 1.74, 95% confidence interval [CI]: 1.41–2.15) and medical and welfare workers (compared with manufacturing, adjusted OR: 1.40, 95% CI: 1.07–1.84). There was also a willingness to undergo HIV testing among public service workers compared with manufacturing (adjusted OR: 1.55, 95% CI: 1.10–2.18). In the company structure category, the presence of occupational health staff at the workplace (compared with their absence) was associated with the desire to undergo HIV testing (adjusted OR: 1.35, 95% CI: 1.16–1.59). Moreover, employees of companies larger than micro scale were more likely to request HIV testing during workplace health checkups. In the implementation of workplace health checkups category, workers who were available for hepatitis testing were more willing to undergo HIV testing than those who were not (adjusted OR: 2.02, 95% CI: 1.66–2.44). In addition, workers who underwent health checkups with a colleague (vs. those who did not) and workers who attended a medical facility for their health checkups (vs. those who underwent health checkups in the workplace) were more willing to take part in HIV testing.

**Table 2:** Occupational factors associated with willingness to undergo voluntary HIV testing during health checkups

**Variable**	**Rate**	**Univariate**			Multivariate*		
		
	**%**	**OR**	**(95% CI)**	***P*-value**	**OR**	**(95% CI)**	***P*-value**
Risk for occupational exposure to blood							
No	25.4	1.00	-	-	1.00	-	-
Unknown	20.5	0.76	(0.43–1.32)	0.322	0.68	(0.38–1.21)	0.185
Yes	41.6	2.09	(1.72–2.54)	<0.001	1.74	(1.41–2.15)	<0.001
Industry							
Manufacturing	27.3	1.00	-	-	1.00	-	-
Retail and wholesale	25.0	0.89	(0.68–1.16)	0.381	0.93	(0.71–1.23)	0.608
Medical and welfare	35.0	1.44	(1.11–1.85)	0.005	1.40	(1.07–1.84)	0.015
Transportation	23.6	0.83	(0.59–1.16)	0.271	0.85	(0.60–1.21)	0.366
Information technology	32.6	1.29	(0.93–1.79)	0.128	1.31	(0.93–1.83)	0.125
Public service	37.0	1.57	(1.13–2.17)	0.007	1.55	(1.10–2.18)	0.012
Education and learning support	25.7	0.92	(0.65–1.32)	0.660	1.03	(0.71–1.50)	0.869
Construction	21.6	0.73	(0.50–1.08)	0.113	0.80	(0.54–1.19)	0.272
Finance	28.8	1.08	(0.74–1.58)	0.688	1.19	(0.80–1.76)	0.387
Catering and hospitality	26.3	0.95	(0.64–1.41)	0.801	0.98	(0.65–1.47)	0.919
Living-related services and entertainment	27.7	1.02	(0.69–1.51)	0.906	1.06	(0.70–1.58)	0.790
Other	23.4	0.82	(0.65–1.02)	0.074	0.86	(0.68–1.08)	0.201
Occupational health staff in the workplace							
No	25.3	1.00	-	-	1.00	-	-
Unknown	20.4	0.76	(0.60–0.96)	0.019	0.70	(0.55–0.89)	0.003
Yes	32.8	1.44	(1.24–1.67)	<0.001	1.35	(1.16–1.59)	<0.001
Company size (number of employees)							
1–9	22.8	1.00	-	-	1.00	-	-
10–49	28.7	1.36	(1.08–1.72)	0.009	1.30	(1.02–1.66)	0.031
50–999	26.5	1.22	(0.99–1.51)	0.063	1.08	(0.87–1.35)	0.476
≥1000	29.9	1.44	(1.16–1.79)	0.001	1.25	(1.00–1.57)	0.055
Voluntary hepatitis B testing during health checkups							
No	25.3	1.00	-	-	1.00	-	-
Unknown	21.1	0.79	(0.66–0.95)	0.014	0.76	(0.63–0.92)	0.005
Yes	43.3	2.26	(1.89–2.70)	<0.001	2.02	(1.66–2.44)	<0.001
Health checkups with colleagues							
No	23.8	1.00	-	-	1.00	-	-
Unknown	19.4	0.77	(0.53–1.12)	0.17	0.65	(0.44–0.96)	0.030
Yes	32.3	1.53	(1.33–1.76)	<0.001	1.42	(1.23–1.64)	<0.001
Location of health checkups							
Workplace	26.9	1.00	-	-	1.00	-	-
Medical facility	28.6	1.09	(0.93–1.27)	0.285	1.19	(1.02–1.39)	0.032
Other	27.3	0.70	(0.54–0.91)	0.009	0.71	(0.54–0.94)	0.015

HIV: human immunodeficiency virus; OR: odds ratio; CI: confidence interval.

*Adjusted for age, sex, marital status, education, and history of HIV testing.

## Discussion

The current study evaluated workplace factors associated with willingness to undergo HIV testing during workplace health checkups. Approximately 27% of participants were willing to undergo HIV testing, which is a decrease from the 41% reported in a previous Japanese study [8]. The decrease in willingness to undergo testing may have been caused by a loss of interest in HIV testing as a result of the COVID-19 pandemic [22]. The current study found that workplace factors that increased the desire for HIV testing during workplace health checkups included occupations with a risk of occupational blood exposure (including medical and welfare roles), the presence of occupational health staff, and larger company size and the resources to implement workplace health checkups (including the provision of hepatitis testing). Pilot HIV testing during workplace health checkups that is similar to existing testing for hepatitis B/C virus and rubella antibodies, starting with workplaces that already comply with the factors we identified in the current study, would facilitate the implementation of HIV testing in the workplace. When carrying out pilot HIV testing, it is essential to establish a comprehensive protocol for managing health information, while also ensuring the protection of privacy and adherence to pertinent laws [23].

In the present study, we found that workers who were at risk of occupational blood exposure and those in the medical and welfare professions were more willing to be tested for HIV in the workplace. This finding is consistent with a previous study, which showed that healthcare workers are typically concerned about their HIV status and desire to undergo HIV testing [24]. Fear of contracting HIV from HIV patients is thought to increase concern among healthcare workers [25, 26]. In addition to healthcare workers, people who are willing to be tested for HIV are likely to have better risk perception of HIV infection and be more informed about HIV [27–29]. Thus, the implementation of a future program of HIV testing during workplace health checkups should involve a pilot study of workers at risk of occupational blood exposure, such as those in medical and welfare roles. Discrimination and stigma against HIV-infected healthcare workers does exist; therefore, the HIV test results obtained from these workplace tests will require careful management [30].

Employees of companies with occupational health staff in the workplace were more likely to want HIV testing during health checkups. Moreover, the employees of companies that were larger than micro scale were more willing to undergo workplace HIV testing. No previous studies have evaluated the relationship between willingness to undergo HIV testing and the presence of occupational health staff or company size. However, it is widely accepted that the presence of occupational health staff can enhance workers’ motivation to undergo HIV testing during workplace health assessments by providing health education and acting as health counselors. These findings could be explained by occupational health staff building a good relationship with company employees and adequately managing their clinical information [31, 32]. Lack of knowledge and awareness about HIV are reported to be barriers to the successful introduction of HIV testing in the workplace [33]. A likely reason for workers in larger companies to be more willing to undergo workplace HIV testing is the presence of occupational health staff in these companies. Further research is warranted on the contextual factors surrounding the presence of occupational health personnel to facilitate the promotion of HIV testing.

The results of the current study suggest that implementing hepatitis testing during health checkups in the workplace could lead to concurrent HIV testing. However, no previous studies have evaluated the relationship between hepatitis and HIV testing in the workplace. Concurrent testing for HIV and other sexually transmitted diseases is crucial for promoting HIV testing [34]. Therefore, simultaneously testing for hepatitis and HIV in the workplace could also be an effective strategy. For example, most workplaces that conduct hepatitis testing already have established frameworks to handle test results in a way that protects patients’ privacy [35]. In addition, concurrent testing may encourage the uptake of HIV testing by reducing the need for additional blood sampling, which is an invasive procedure [36]. Future research should be conducted to evaluate the effectiveness of concurrent hepatitis and HIV testing during workplace health checkups.

In the current study, individuals who were willing to undergo HIV testing during workplace health checkups tended to work in public services and attended health checkups together with their colleagues at a medical facility. Although the reasons underlying these findings are not entirely clear, the category of public service contains diverse occupations, and those who have more knowledge about HIV, such as public health workers and national and local government officials, may be more inclined to undergo HIV testing. Further research is needed to elucidate the mechanisms underlying the effects of these workplace factors.

The present study had several limitations that should be considered. First, this was a cross-sectional study, making it impossible to determine cause-effect relationships. Second, this study was conducted via the Internet, meaning that we only gathered data from respondents who had access to the Internet and therefore the results are not representative of the Japanese population (selection bias). For example, respondents may have been more informed about HIV infection than individuals who do not use the Internet [37].

Thus, the present results cannot be accurately extrapolated to members of the population without Internet access. However, because some of the information gathered is sensitive (e.g., prior history of HIV testing), an Internet survey is considered to be an appropriate sampling method. Finally, in this survey, we did not ask respondents whether they were at high risk of HIV infection, such as men who have sex with men, people who have sex with commercial sex workers, or people who inject drugs. These high-risk individuals may be more likely to wish to undergo HIV testing.

## Conclusions

This study evaluated workplace factors associated with willingness to undergo HIV testing during workplace health checkups. We found that workers who were at risk of occupational blood exposure and those in medical and welfare occupations were more willing to undergo HIV testing compared with other groups. Similarly, many workers desired HIV testing if the company had occupational health staff present and offered workplace health checkups that included hepatitis testing. To better implement HIV testing in workplace health checkups in the future, we recommend conducting a pilot study involving people at risk of occupational blood exposure (i.e., medical and welfare workers) and employees of companies with occupational health staff.

## Abbreviations

HIV: human immunodeficiency virus
COVID-19: coronavirus disease 2019
AIDS: acquired immunodeficiency syndrome
